# Carbon fluoroxide nanoparticles as fluorescent labels and sonosensitizers for theranostic applications

**DOI:** 10.1088/1468-6996/16/4/044601

**Published:** 2015-07-16

**Authors:** Alexander Kharin, Olga Syshchyk, Alain Geloen, Sergey Alekseev, Andrey Rogov, Vladimir Lysenko, Victor Timoshenko

**Affiliations:** 1 University of Lyon, CarMeN Laboratory, UMR INSERM 1060, INSA de Lyon, University of Lyon, France; 2 Institute of High Technologies, Taras Shevchenko National University of Kyiv, 64, Volodymyrs’ka St., 01601 Kyiv, Ukraine; 3 Chemistry Faculty, Taras Shevchenko National University of Kyiv, 64, Volodymyrs’ka St., 01601 Kyiv, Ukraine; 4 GAP-Biophotonics, University of Geneva, 22, ch. de Pinchat, CH-1211 Geneva 4, Switzerland; 5 University of Lyon, Nanotechnology Institute of Lyon (INL) UMR 5270, CNRS, INSA Lyon, Villeurbanne, F-69621, France; 6 Moscow State Lomonosov University, Physics Department, Leninskie Gory 1, 119991 Moscow, Russia

**Keywords:** nanoparticles, theranostics, ultrasound, carbon, luminescence, bioimaging

## Abstract

Carbon fluoroxide (CFO) nanoparticles (NPs) produced from silicon carbide wafers are used as both fluorescent probes and sonosensitizers for theranostic application. *In vitro* cell tests were carried out to investigate the feasibility of ultrasound-based therapy with the use of the CFO NPs. The NPs that penetrated inside the cells were shown to provoke cell destruction after application of an ultrasound treatment. No significant toxic effect was observed when the cells were treated with NP concentrations up to 0.5 mg ml^−1^ without applying ultrasound treatment. The obtained results open a new way toward cancer therapy strategies.

## 1. Introduction

Theranostics is an interdisciplinary field with simultaneous integration of diagnosis and therapy actions [1]. Its main purpose is to diagnose and treat the diseases at their earliest stage, when the diseases are most likely curable or at least treatable. Theranostics often refers to molecular/macromolecular targeting vectors and nano-platform technologies incorporating both diagnostic and therapeutic functionalities. This bifunctionality is one of the key features of a theranostic agent or action.

The most commonly used diagnostic modalities are optical, magnetic resonance imaging (MRI), positron emission tomography, computed tomography, single-photon emission computed tomography, and ultrasound [2]. Optically based imaging is the most cost-effective diagnostic method, but its most serious drawback is related to very short penetration length of the exciting and emitted light signals. A possible solution to this problem can be the use of two-photon excitation in the red/near-infrared (IR) spectral range, where biological tissues have low absorption and scattering is less severe. A therapeutic treatment can be carried out in various ways: chemo- and radio-therapy [3], hyperthermia [4], photodynamic therapy [5], photoacoustics [6], non-linear optics [7], and others. One of the recently developed innovative methods to be applied for a therapeutic purpose is to kill cancer cells by means of ultrasound waves with relatively high intensity. In particular, porous submicrometer- and micrometer-sized particles have been successfully used as sonosensitizers to additionally enhance the ultrasound action [8]. There are two main mechanisms related to the ultrasound-assisted therapeutic action of such nanoparticles (NPs): the first one is a hyperthermia effect, which is maximal in the case of comparable ultrasound wavelength and particles’ size, and the second one is the mechanical damage, which is the maximal case of maximal penetration of the particles into the cells.

There are several opportunities to provide the necessary bi-functionality of a theranostic agent. Some drug delivery techniques can be used to unite different compounds in a single agent [9, 10]. This way is widely used, but the complex nature of the agent requires that the drug, the imaging component, and a carrier are linked together so that the resulting drug delivery system gets the sizes close to tenths or even hundreds of nanometers. Another method is to use the same property, for example, radioactivity both for cancer monitoring and therapy. The third method is based on different properties of a single compound. NPs correspond to the last type of theranostic approaches. For example, iron oxide NPs are agents for MRI, and they can be also used as therapeutic agents for magnetic-field-induced hyperthermia. The main problem of almost any kind of inorganic NPs (such as metal, A^II^B^VI^, A^III^B^V^, and others) is their relatively high toxicity. The least toxic inorganic NPs and quantum dots belong to the IVth group of the periodic table.

Group IV NPs have emerged as very promising nanomaterials for biological applications, such as imaging and drug delivery [11, 12]. They have gradually evolved into promising biomaterials due to their natural biocompatibility; no toxic ions are released when they are used *in vitro* or *in vivo*, and their wide fluorescence spectra span the near-IR, visible, and near-(UV) ranges. Additionally, they are characterized by enhanced resistance to the photobleaching effect and photoluminesence (PL) lifetimes from nanoseconds to microseconds, which are quite suitable for bioimaging purposes. Carbon nanomaterials have attracted particular interest in biomedicine, owing to their highly enriched, distinctive physical and chemical properties [13]. A specific class of carbon nanomaterials termed fluorescent carbon NPs with tunable emission are considered to be next-generation green nanomaterials and are promising alternatives to fluorescent semiconductor nanocrystals due to their high water solubility, flexibility in surface modification with various chemicals, excellent biocompatibility, good cell permeability, and high photostability [14]. On the basis of their synthesis, these particles may contain different functional groups on their surface, namely, –COOH, –OH, >CO, and –NH_2_, which impart excellent water solubility and possibilities for covalent conjugation with the chemotherapeutic agent, targeting agent, and/or antibody. For example, carbon dots with oligomeric polyethylene glycol diamine as the surface functionalization agent were shown to be nontoxic and amenable to fluorescence bioimaging applications *in vitro* and *in vivo* [15]. A chemotherapeutic drug in combination with a targeting agent could be easily tethered to the carbon NPs through covalent linkage with these functional groups. Development of highly biocompatible and fluorescent drug delivery systems based on fluorescent carbon NPs holds great promise for specific drug delivery with minimal side effects and toxicity in cancer patients and provides valuable tools to medicinal chemists for the synthesis of site-specific carriers of various therapeutic agents, with possible application also as imaging systems [16].

In this paper, new carbon fluoroxide (CFO) NPs are used as very efficient theranostic agents both for one- and two-photon excited luminescence cell imaging and as sonosensitizers for ultrasound-assisted therapy.

Since the NPs can be used as both fluorescent probes and sonosensitizers, they are very attractive agents for the imaging and therapy of cancer. As has been shown previously [17], localization of the NPs inside the cells depends on their surface charges. Thus, they give us a unique opportunity to check how they will act as sensitizers being localized in different parts of the cells. Indeed, if the main therapeutic factor is related to the destruction of chromosomes, the NPs accumulated inside the nuclei will ensure the strongest killing effect compared to those accumulated into the cytoplasm. Fluorescence of the NPs allows non-destructive estimation of their localization before the ultrasound treatment.

## 2. Synthesis and characterization of nanoparticles

**Figure 1.**
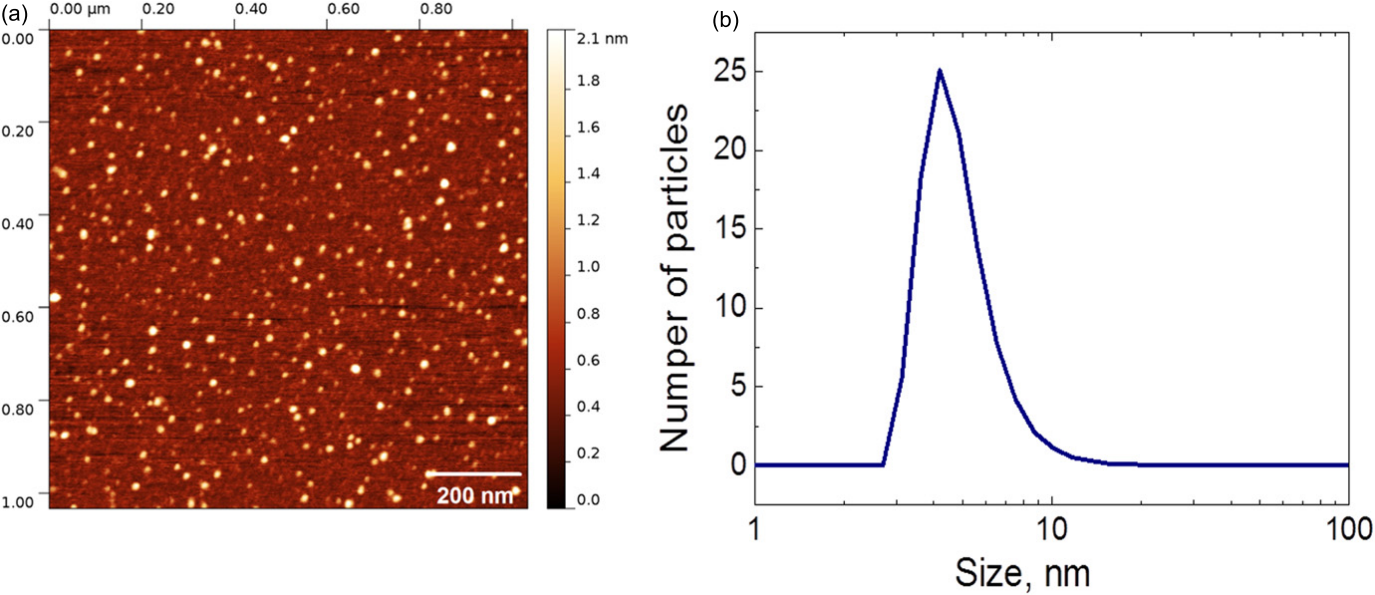
(a) Atomic-force microscopy image of CFO NPs; (b) size distribution of the CFO NPs measured by means of dynamic light scattering.

**Figure 2.**
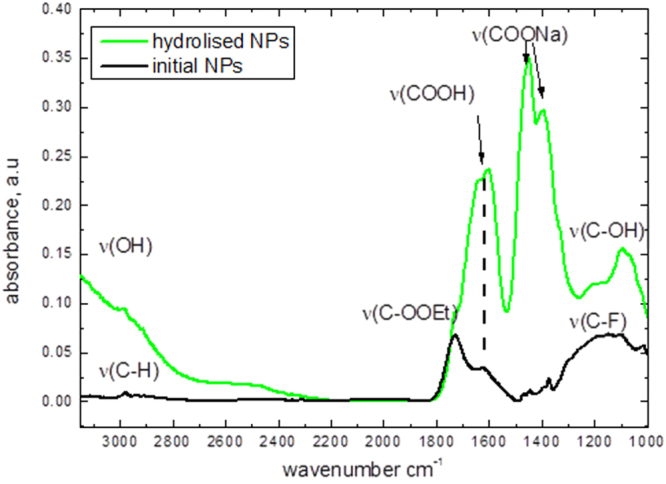
Fourier transform infrared spectrum of the CFO NPs.

Fluorescent CFO NPs were formed by means of electrochemical anodization of a low-resistivity grade (<1 *Ω*.cm) bulk 3C-SiC polycrystalline wafer. The etching process took place for 3 h at a current density of 25 mA cm^−2^ using a 1:1 HF (50%)/ethanol electrolyte. The prepared porous layer was dried in air for several hours. The dried layer was removed from the SiC wafer and then mechanically ground and dispersed in a Krebs buffer solution. The formed colloidal suspension was centrifuged at 10 000 × g for 5 min in order to collect only its top part, containing very small (<10 nm) and homogeneously dispersed NPs, which can be visualized on an atomic force microscopy image, as shown in figure 1(a). As one can see, the particles are not agglomerated and have a spherical-like shape. Size distribution of the obtained NPs was estimated from dynamic light scattering measurements (see figure 1(b)), and the average NP size is found to be in the range of 4–6 nm. The Fourier transform IR spectrum shown in figure 2 gives an idea about dominant chemical bonds (C–H, C=O, and C–O), taking place in the fabricated NPs. Thus, the NP’s surface is supposed to be mainly covered by carboxylic and ester groups.

The shown IR spectra have been obtained for: (i) the dried initial CFO NPs and (ii) the NPs dried after their hydrolysis in NaOH solution with pH 13 to evaluate surface group evolution after the hydrolysis. Some vibrations corresponding to the presence of carboxylic groups at the NP surface have been found. After hydrolysis, they turn into carboxylic salt groups. C–F stretch vibration groups are also present at 1000–1200 cm^−1^. This peak decreases after hydrolysis in a strong base with exchanging C–F groups by C–OH ones (wide band centered at 3000 cm^−1)^. To conclude, the surface chemistry is mainly represented by carboxylic groups, while a small amount of the ethyl ester and C–F groups is also observable.

**Figure 3.**
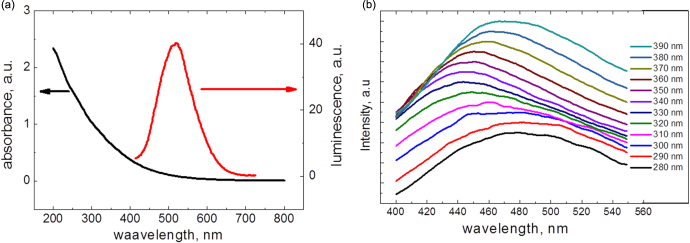
(a) Absorbance and one-photon-excited (excitation wavelength: 400 nm) luminescence spectra of the CFO NPs dispersed in Krebs buffer solution at 0.1 mg ml^−1^ concentration; (b) excitation dependence of the CFO nanoparticles fluorescence.

In addition, an elemental composition of the NPs detailed in table 1 can be described by the C_100_H_104.1_F_19.5_O_51_ gross formula. Such composition can correspond to fluorinated grapheme oxide, but it has the different optical properties [18]. Absorbance and one-photon excited luminescence spectra of the CFO NPs are shown in figure 3. As one can see, the CFO NPs have a strong absorbance in the UV spectral range, and their photoluminescence, excited at 400 nm, is centered near 550 nm. The shape of the spectra is excitation-dependent, so we can propose the presence of different emission centers, which are excited preferentially by the different wavelengths.

**Table 1.** Elemental composition of the CFO NPs.

Element	C	H	F	O
Atomic %	36.5	38	7	18.5

## 3. Results and discussion

3T3-L1 (fibroblasts) and HuH7 (hepatocarcinoma) cells were grown in Dulbecco’s modified Eagle’s medium supplemented with 10% newborn calf serum 100 IU penicillin, 0.1 mg streptomycin, and 0.25 mg L^−1^ amphotericin B at 37 °C in a water-saturated atmosphere, with 5% CO_2_ in air, in a Heraeus incubator. The cells were trypsinized, and about 2500 cells were added to each well in a 96-well plate for the cell proliferation measurements. Then the cells were incubated for 48 h. CFO NPs with a concentration of 0.25 mg ml^−1^ were added to the cell cultures, which were additionally incubated for 24 h. Before acquisition of a fluorescent image of the cells, the NPs were washed out from the extracellular environment with phosphate-buffered saline.

**Figure 4.**
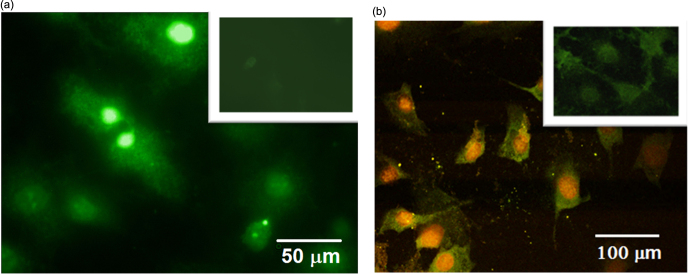
Fluorescence microscopy images of the cells labeled with CFO NPs: (a) one-photon excited fluorescence microscopy, excitation: 490 nm, emission: 525 nm; (b) two-photon excited fluorescence microscopy, excitation: 790 nm, emission channels: green (480–490 nm), yellow (500–550 nm), red (610–630 nm). Control cell lines without NPs are shown as inserts.

Figure 4(a) shows a one-photon excited fluorescence image of labeled 3T3-L1 fibroblast cells obtained by means of a Leica DMI 4000B microscope with the following filter combination: UV/violet excitation band: 354–424 nm and observation spectral range: >470 nm. One can easily notice a heterogeneous distribution of the fluorescence intensity inside the labeled cells. Indeed, preferential localization of the fluorescent CFO NPs in the cell nuclei ensures their very bright visualization. Since the UV excitation cannot penetrate sufficiently deeply into biological tissues, near-IR excitation and a Nikon A1R-MP nonlinear microscope were used for the multi-channeled imaging of two-photon-excited fluorescence (see figure 4(b)). The preferential accumulation of the CFO NPs in the cell nuclei is confirmed once again. Indeed, the higher local concentration of the NPs in the cell nuclei leads to their red-shifted luminescence in comparison with the lower-concentrated NPs in the cell cytoplasm. It is worth remarking that the incorporation of the NPs into the cells does not lead to any significant changes of the cell dimension and shape.

**Figure 5.**
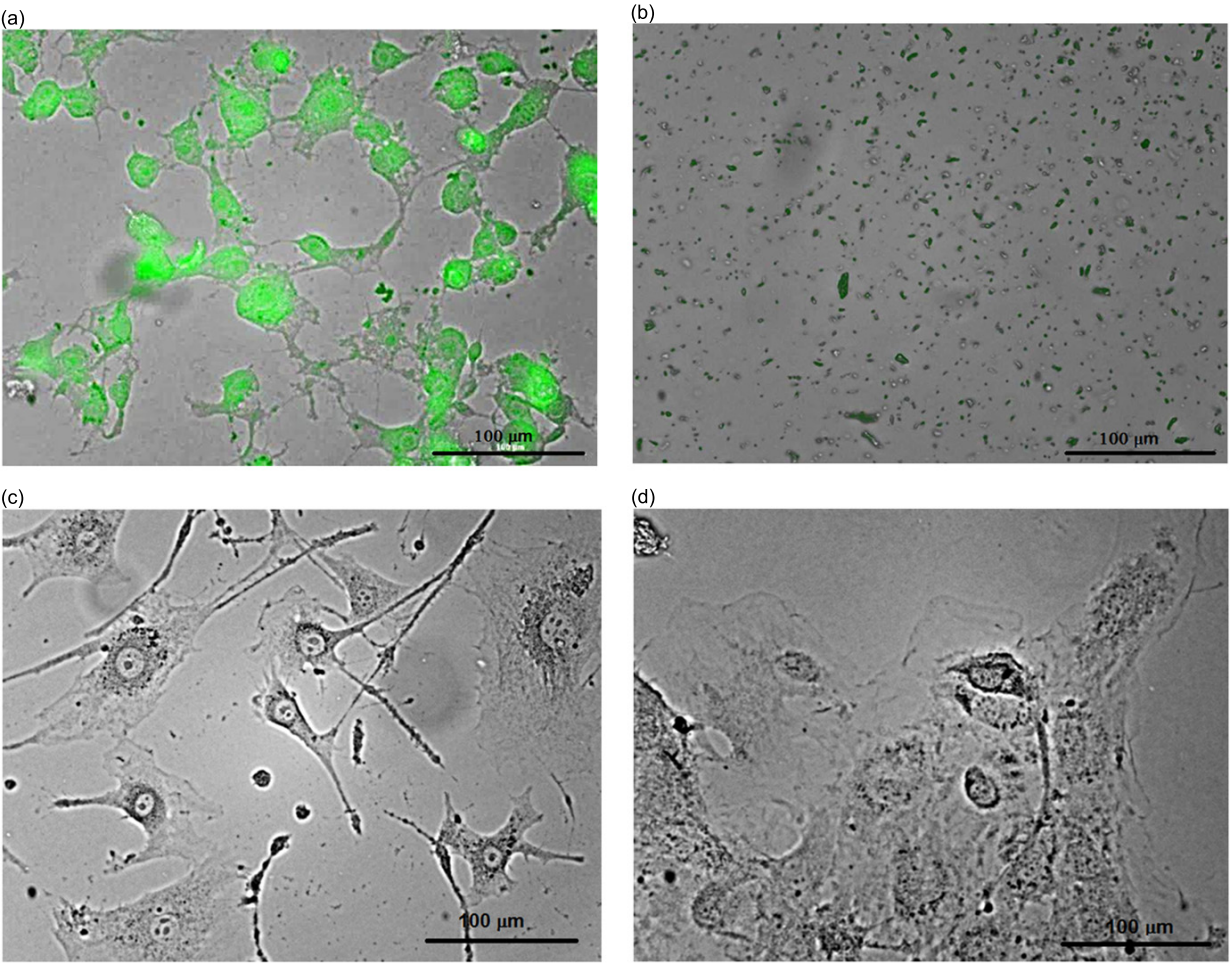
3T3-L1 cells labeled with CFO NPs (1 mg ml^−1^) before (a) and after (b) ultrasound treatment. Non-labeled 3T3-L1 cells before (c) and after (d) ultrasound treatment are shown for comparison.

As illustrated in figure 5, ultrasound treatment (40 kHz, power density: 0.4 W cm^−3^, time: 10 min) of the cell cultures labeled with the luminescent CFO NPs leads to their complete destruction (figures 5(a) and (b)), while the non-labeled cells remain undamaged (figures 5(c) and (d)). Thus, the CFO NPs are shown to be able to play a double role of one- or multi-photon excited fluorescent labels as well as ultrasonic sensitizers, selectively destroying only the labeled cells.

**Figure 6.**
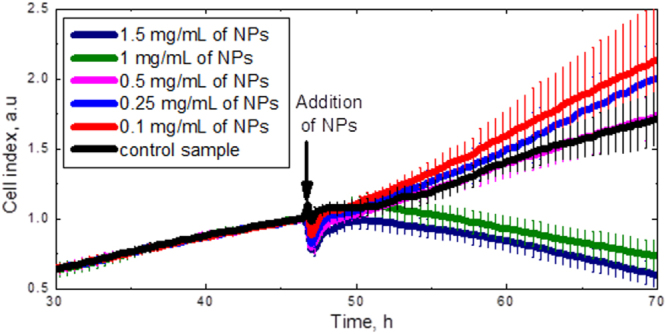
Dose response curves of the 3T3-L1 cell numbers. The arrow shows the times when the CFO NPs were added.

The toxicity of the CFO NPs can be reflected as time evolution of the proliferating cell numbers, which was studied by means of the xCelligence system. Figure 6 shows the time dependence of the cell index, which is directly proportional to the cell number. The NPs with different concentrations were added to the cells at the 48th hour. As can be clearly seen, the NP concentrations below 0.5 mg ml^−1^ are not toxic for the 3T3-L1 cells, since their proliferation dynamics is very similar to the control cell lines without NPs. Thus, the concentration value of 0.25 mg ml^−1^ was definitely chosen for the following experiments.

**Figure 7.**
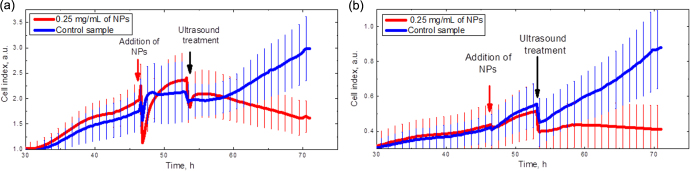
Time evolution of the cell number: (a) 3T3-L1 cell line; (b) HuH7 cancer cell line. Arrows show when the CFO NPs were added and ultrasound treatment was switched on.

In order to check the effects of NP toxicity as well as of ultrasound treatment on the labeled and non-labeled cells, time evolution of the cell numbers was studied by means of the xCelligence system. Figure 7 shows the time dependence of the cell index. Arrows indicate the moments when the CFO NPs are added to cells and ultrasound treatment is switched on for 10 min. Red lines correspond to the cells labeled with the fluorescent CFO NPs, and blue ones to control cell lines (without NPs). As one can see, the addition of the NPs to the 3T3-L1 cell line leads to an instant decrease of the cell index with relatively fast relaxation (see figure 7(a)). This can be caused by cell shrinking. After relaxation, the cells restore their initial shape. After ultrasound treatment, the number of the labeled 3T3-L1 cells goes down rapidly because of their significant destruction described above, while the proliferation velocity of a control (non-labeled) cell line is completely restored. Similar effects are observed in the case of cancer cells (figure 7(b)). Thus, one can conclude that the NPs and ultrasound treatment alone have no toxic or damaging effect on the cells, while their combined action provokes significant cell injury, which can be efficiently used for theranostic purposes.

## 4. Conclusion

The reported *in vitro* cell tests were carried out to investigate the feasibility of ultrasound-assisted (40 kHz) treatment for cancer therapy based on the use of the CFO NPs. In particular, the ultrasound treatment of cell lines labeled with fluorescent NPs leads to cell death, in contrast to the non-labeled cells. The cell lines labeled with an increased concentration of the CFO NPs can even provoke a complete destruction of the cells under the ultrasound treatment. However, an *in vivo* systematic administration of cancers still remains an important challenge for this therapeutic approach. Some experiments on this issue are under investigation, using tumor-targeting techniques.
